# Development and validation of a novel prognostic nomogram for advanced diffuse large B cell lymphoma

**DOI:** 10.1007/s10238-024-01326-y

**Published:** 2024-03-30

**Authors:** Mengdi Wan, Wei Zhang, He Huang, Xiaojie Fang, Yungchang Chen, Ying Tian, Yuyi Yao, Huawei Weng, Zegeng Chen, Le Yu, Yuke Tian, Huageng Huang, Xudong Li, Huangming Hong, Tongyu Lin

**Affiliations:** 1 Department of Medical Oncology, Sichuan Cancer Hospital and Institute, Sichuan Cancer Center, University of Electronic Science and Technology of China, Chengdu, 610054 Sichuan Province China; 2 Department of Medical Oncology, State Key Laboratory of Oncology in Southern China and Collaborative Innovation Center of Cancer Medicine, Sun Yat-Sen University Cancer Center, 651 Dongfeng, Road East, Guangzhou, 510060 Guangdong China

**Keywords:** DLBCL, NHL, Nomogram, Prognosis

## Abstract

Advanced diffuse large B cell lymphoma (DLBCL) is a common malignant tumor with aggressive clinical features and poor prognosis. At present, there is lack of effective prognostic tool for patients with advanced (stage III/IV) DLBCL. The aim of this study is to identify prognostic indicators that affect survival and response and establish the first survival prediction nomogram for advanced DLBCL. A total of 402 patients with advanced DLBCL were enrolled in this study. COX multivariate analysis was used to obtain independent prognostic factors. The independent prognostic factors were included in the nomogram, and the nomogram to predict the performance of the model was established by R rms package, C-index (consistency index), AUC curve and calibration curve. The training and validation cohorts included 281 and 121 patients. In the training cohort, multivariate analysis showed that Ki-67 (70% (high expression) vs ≤ 70% (low expression), *p* < 0.001), LDH (lactate dehydrogenase) (elevated vs normal, *p* = 0.05), FER (ferritin) (elevated vs normal, *p* < 0.001), and β2-microglobulin (elevated vs normal, *p* < 0.001) were independent predictors and the nomogram was constructed. The nomogram showed that there was a significant difference in OS among the low-risk, intermediate-risk and high-risk groups, with 5-year survival rates of 81.6%, 44% and 6%, respectively. The C-index of the nomogram in the training group was 0.76. The internal validation of the training group showed good consistency. In the internal validation cohort of the training group, the AUC was 0.828, and similar results were obtained in the validation group, with a C-index of 0.74 and an AUC of 0.803. The proposed nomogram provided a valuable individualized risk assessment of OS in advanced DLBCL patients.

## Introduction

DLBCL is the most common type of non-Hodgkin's lymphoma, accounting for about 30–40% of all non-Hodgkin's lymphomas [1], with strong heterogeneity and poor prognosis [2, 3]. According to the Ann Arbor staging system, about 60–70% of DLBCL patients have stage III/IV at the time of initial diagnosis and advanced stage is important factor that indicating poor prognosis [4, 5]. The 10-year overall survival (OS) rate of advanced DLBCL is typically 43% [6]. Although 50–60% of DLBCL patients can be cured by the current first-line R-CHOP(rituximab, cyclophosphamide, doxorubicin, vincristine and prednisolone) regimen [7–9], about 40% of patients are still at risk of disease recurrence [10–12] and the prognosis of patients with advanced DLBCL is still poor [13]. For patients with advanced DLBCL, the first-line treatment is usually R-CHOP or R-DA-EPOCH (Rituximab, etoposide, vindesine, epirubicin, cyclophosphamide, prednisone) [14] chemotherapy [5, 15]. For initial large tumors (>7.5 cm) is usually followed by radiotherapy [13, 16]. In addition, patients with advanced DLBCL are also more likely to experience treatment failure than those with early-stage DLBCL [17]. Approximately 90% of early-stage DLBCL patients achieved long-term disease control with R-CHOP, compared with only 60% of advanced DLBCL patients [5].

Since the 1990s, the IPI score constructed from disease stage, age, serum lactate dehydrogenase (LDH) concentration, Eastern Cooperative Oncology Group (ECOG), performance status (PS), and the number of extranodal sites has been a standard tool for risk stratification and treatment guidance [18]. In the IPI score, stage is an important prognostic factor, and the higher the stage, the worse the prognosis [19]. However, there is no prognostic tool for advanced-stage DLBCL, and the means to guide precise treatment are limited. Therefore, advanced-stage DLBCL urgently needs a good prognostic tool to guide treatment.

Unlike traditional models such as IPI and R-IPI, the nomogram is a visualized statistical predictive model that identifies points of value for each variable [20], thereby improving the predictive accuracy of clinical outcomes. Previously, nomogram was demonstrated in DLBCL as well as in several malignancies, showing accurate estimates of patient survival [21, 22]. The aim of this study is to evaluate the clinical characteristics, identify the prognostic indicators that affect the survival and efficacy, and establish the first survival prediction nomogram for advanced DLBCL, which is compared with the traditional IPI prognostic system.

## Patients and methods

Patients with advanced DLBCL who were initially treated in 12 hospitals (Sichuan Province Cancer Hospital, Sun Yat-sen University Affiliated Cancer Hospital, Hunan Province Cancer Hospital, Guizhou Province Cancer Hospital, Fujian Province Cancer Hospital, Jiangxi Province Cancer Hospital, Yunnan Province Cancer Hospital, Liuzhou People's Hospital, the First People's Hospital of Yunnan Province, Mianyang Central Hospital, the Sixth Affiliated Hospital of Sun Yat-sen University, Henan Province Cancer Hospital.) from February 2012 to November 2022 were collected. The inclusion criteria for this study were as follows: Patients with histologically confirmed DLBCL, the Ann Arbor stage of lymphoma was stage III/IV (advanced), and complete clinical data were enrolled. Staging procedures include medical history, physical examination, hematology (including blood routine, biochemical routine, blood calcium, LDH, etc.), neck, chest, abdomen, pelvis enhanced CT or whole body PET-CT, bone marrow puncture smear and biopsy, and add gastroenteroscopy to those considered to have gastrointestinal band invasion. Ann Arbor staging was followed: invasion of a single lymph node region (I) or a single external node (I E). Invasion of more than 2 lymph node regions, but both in the same side of the diaphragm (II), may be accompanied by ipsilateral localized extranodal organ invasion (IIE). Both the upper and lower lymph node regions of the diaphragm are invaded (III), and may be accompanied by localized extranodal organ invasion (IIIE) or splenic invasion (IIIS), or both (IIIES). Diffuse, disseminated extranodal organ or tissue invasion, with or without lymph node invasion (stage IV). The clinical data included sex, age, extranodal sites, bulky (the size of the primary tumor was bigger than 7.5 cm), cell of origin subtypes, first-line chemotherapy regimens, BCL-2, BCL-6, C-MYC, CD10, CD5, Ki-67, hepatitis B antigen, LDH, Alb (Albumin), PLT (Blood platelet), HB (Hemoglobin), FER, and β2 microglobulin. After excluding patients who lacked standard treatment (such as R-CHOP) cases and were younger than 18 years old, 402 patients who met the selection criteria were included in the analysis and entered the nomogram study cohort. Expression of biomarkers CD5, MYC, BCL-2, BCL-6, and Ki-67 was assessed using the respective antibodies. All histopathological slides were confirmed by at least 2 expert pathologists. The cut-off points of MYC, BCL-2 and BCL-6 protein were 40%, 50% and 50% positive staining of lymphoma cells, respectively. GCB or non-GCB phenotypes were determined according to the Hans algorithm. We randomly assigned 281 patients, in a 7:3 ratio, to the training group and 121 patients to the validation group.

### Clinical indicators

Clinical characteristics assessed by clinical indicators include baseline characteristics (sex, age, extranodal sites, ECOG, bulky, cell of origin subtypes, first-line chemotherapy regimens, BCL-2, BCL-6, C-MYC, CD10, CD5, Ki-67, hepatitis B antigen, LDH, Alb, PLT, HB, FER, and β2 microglobulin). Response was assessed according to the International Working Group criteria. OS was defined as the survival time from initial diagnosis to the last follow-up or death.

### Nomogram construction and validation

First, univariate Cox regression analysis was performed in the training group to screen out factors related to OS of patients with advanced DLBCL, and then multivariate Cox analysis was used to obtain independent prognostic factors affecting OS of patients with advanced DLBCL, which were then applied to the development of the nomogram. Internal validation was first performed, estimating the C-index by analyzing the area under the curve (AUC) of the receiver operating characteristic (ROC) curve. Next, we constructed a calibration plot to determine whether the predicted and observed survival probabilities agree. This figure uses Bootstrap resampling (1000 resamples). Finally, we performed external validation in which the nomogram was used to evaluate each patient in the validation cohort and Cox regression analysis was performed using the total score of each patient as an independent factor. Regression analysis was then used to derive the C-index and calibration curve.

### Statistical analysis

Survival curves were estimated by the Kaplan–Meier method and compared with log-rank tests stratified by prognostic factors. Nomogram was constructed based on Cox model parameter estimates in the training cohort. The selection of the final model was performed using a reverse stepwise descent selection procedure. Nomogram was constructed and validated according to Iasonos guidelines [20]. Statistical analyses were performed using the Hmisc, rms, survival ROC package in IBM SPSS Statistics (version 20.0) and R (version 4.2.1) (http://www.r-project.org/).

## Result

### Patient characteristics

This study included 402 patients with newly diagnosed stage III/IV DLBCL. Table 1 shows the clinical characteristics of 281 patients with stage III/IV DLBCL in the training group. The median survival time in the training group was 55 months. A total of 263 (65.4%) patients survived and 139 (34.6%) died, 87 of whom died from disease progression and 37 died from tumor-related complications, including 9 from infection-related disease and 6 from respiratory failure due to tumor compression of the mediastinum. Ten patients died of massive gastrointestinal hemorrhage due to tumor rupture, and 12 died due to cachexia. The final 15 patients died of non-tumor related diseases, including 3 patients who died of cerebral infarction, 4 patients who died of heart failure, 5 patients who died of severe pneumonia, and 3 patients who died of natural causes after recovery. According to MaxStat method, the optimal cut-off values of Age, LDH, Alb, PLT, HB, FER, and β2 microglobulin were 60 years old, 245U/L, 40 g/L, 100*10^9/L, 120 g/L, 130 μg/L, and 3.05 mg/L, respectively. There were 152 males (54.1%) and 129 females (45.9%). A total of 145 cases (51.6%) were less than 60 years old, and 136 cases (48.4%) were more than 60 years old at the first diagnosis. Of all patients in the training group who received first-line treatment, 202 (71.8%) received chemotherapy with R-CHOP, 35 (12.4%) received chemotherapy with CHOP, 13 (4.6%) received chemotherapy with R-DA-EPOCH, and 31 (11.2%) received other treatments. There were 260 cases (92.4%) with ECOG score 0–1 and 21 cases (7.6%) with ECOG score greater than 1. The number of extranodal involvement was 0–1 in 138 (49.1%) patients and 1 or more in 143 (50.9%) patients. There were 79 cases (28.1%) with large tumors at initial diagnosis. There were 134 cases (51.2%) of GCB subtype and 128 cases (48.9%) of non-GCB subtype. A total of 202 patients (72.7%) were treated with R-CHOP as first-line treatment. At initial diagnosis, 215 (76.5%) patients were positive for BCL-2, 232 (82.6%) patients were positive for BCL-6, 216 (76.9%) patients were positive for C-MYC, and 86 (30.6%) patients were positive for CD10. The 260(92.4%) patients had an ECOG score of 0–1. CD5 expression was positive in 92 (32.7%) patients, Ki-67 was low expressed in 75 (26.7%) patients and high expressed in 206 (73.3%) patients. Hepatitis B surface antigen was positive in 41 (14.6%) patients, and LDH was higher than normal value in 148 (52.7%) patients. After first-line treatment, 86 patients (30.6%) achieved CR (Complete remission),71 patients (25.2%) achieved PR(Partial remission), 55 patients (19.6%) achieved SD(Stable disease), and the remaining 69 patients (24.6%) achieved PD(Progressive disease).

**Table 1:** Clinical characteristics of 402 patients with advanced DLBCL

Characteristic	All patients	Training cohort	Validation cohort
Total	402	281	121
*Sex*			
Male	210 (52.2%)	152 (54.1%)	58 (47.9%)
Female	192(47.8%)	129(45.9%)	63(52.1%)
*Age*			
< = 60	207(51.5%)	145(51.6%)	62(51.2%)
> 60	195 (48.5%)	136 (48.4%)	59 (48.8%)
*Extranodal sites*			
0–1	189(47.0%)	138(49.1%)	51(42.1%)
> = 2	213 (53.0%)	143 (50.9%)	70 (57.9%)
*Bulky*			
Negative	282(70.1%)	202(71.9%)	80(66.1%)
Positive	120 (29.9%)	79 (28.1%)	41 (33.9%)
*Cell of origin subtypes*			
GCB	189 (50.1%)	134(51.2%)	55(47.8%)
Non-GCB	188 (49.9%)	128(48.9%)	60 (52.2%)
*BCL-2*			
Negative	96(23.9%)	66(23.5%)	30(24.8%)
Positive	306 (76.1%)	215 (76.5%)	91 (75.2%)
*BCL-6*			
Negative	71(17.7%)	49(17.4%)	22(18.2%)
Positive	331 (82.3%)	232 (82.6%)	99 (81.8%)
*C-MYC*			
Negative	91(22.6%)	65(23.1%)	26(21.5%)
Positive	311 (77.4%)	216 (76.9%)	95 (78.5%)
*CD10*			
Negative	280(69.7%)	195(69.4%)	85(70.2%)
Positive	122 (30.3%)	86 (30.6%)	36 (29.8%)
*CD5*			
Negative	276(68.7%)	189(67.3%)	87(71.9%)
Positive	126 (31.3%)	92 (32.7%)	34 (28.1%)
*Ki-67*			
< = 70%	113(29.1%)	75(26.7%)	38(31.4%)
> 70%	289(71.9%)	206 (73.3%)	83 (68.6%)
*Hepatitis B antigen*			
Negative	340(84.6%)	240(85.4%)	100(82.6%)
Positive	62 (15.4%)	41 (14.6%)	21(17.4%)
*Elevated LDH*			
No	200(49.8%)	133 (47.3%)	67(55.4%)
Yes	202 (50.2%)	148(52.7%)	54 (44.6%)
*Alb*			
< = 40	180(44.8%)	123(43.8%)	57(47.1%)
> 40	222 (55.2%)	158 (56.2%)	64 (52.9%)
*PLT*			
< = 100	32(8.0%)	23(8.1%)	9(7.4%)
> 100	370 (92.0%)	258 (91.9%)	112(92.6%)
*HB*			
< = 120	236(58.7%)	164(58.4%)	72(59.5%)
> 120	166 (41.3%)	117 (41.6%)	49 (40.5&)
*FER*			
< = 130	195(48.5%)	138(49.1%)	57(47.1%)
> 130	207 (51.5%)	143 (50.9%)	64 (52.9%)
*β2 microglobulin*			
< = 3.05	229(57.0%)	162(57.7%)	67(55.4%)
> 3.05	173 (43.0%)	119 (42.3%)	54 (44.6%)
*ECOG*			
0–1	385(95.7%)	260 (92.4%)	125 (88.7%)
> 1	37(9.3%)	21 (7.6%)	16 (11.3%)
*Efficacy evaluation*			
CR	118(29.4%)	86(30.6%)	32(26.4%)
PR	109(27.1%)	71(25.2%)	38(31.4%)
SD	74(18.4%)	55(19.6%)	19(15.8%)
PD	101(25.1%)	69(24.6%)	32(26.4%)

FER, ferritin; Alb, Albumin; PLT, Blood platelet; HB, Hemoglobin; ECOG, Eastern Cooperative Oncology Group; CR, Complete remission; PR, Partial remission; SD, Stable disease; PD, Progressive disease.

### Survival analysis, nomogram construction and internal validation

**Fig. 1:**
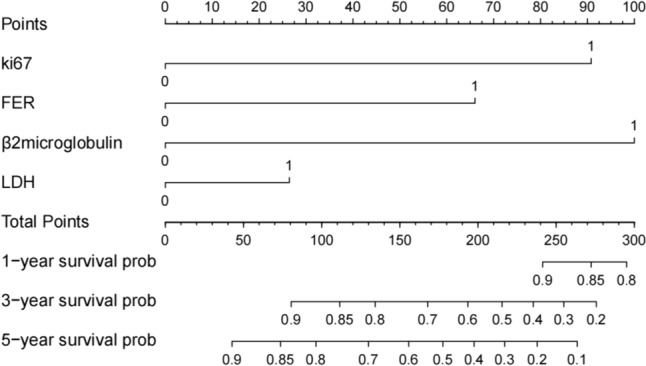
Nomogram model based on patients in the training group

**Fig. 2:**
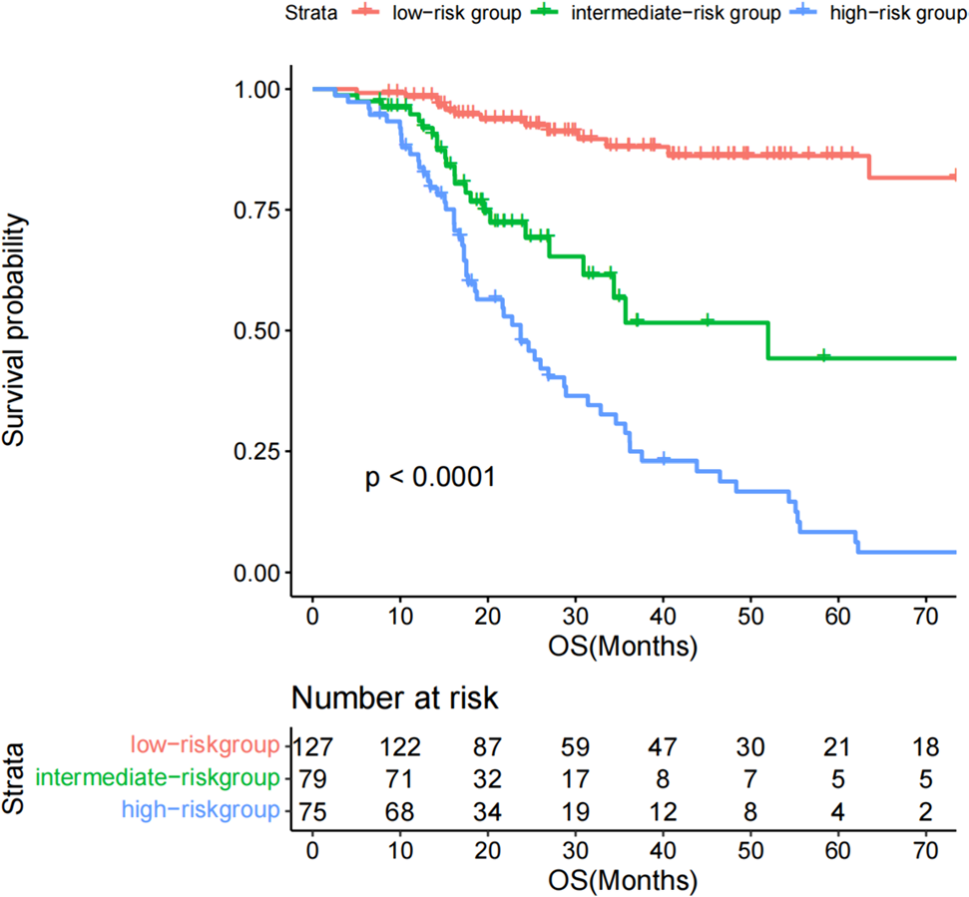
Kaplan–Meier survival curves for risk groups of the nomogram model in the training group

**Fig. 3:**
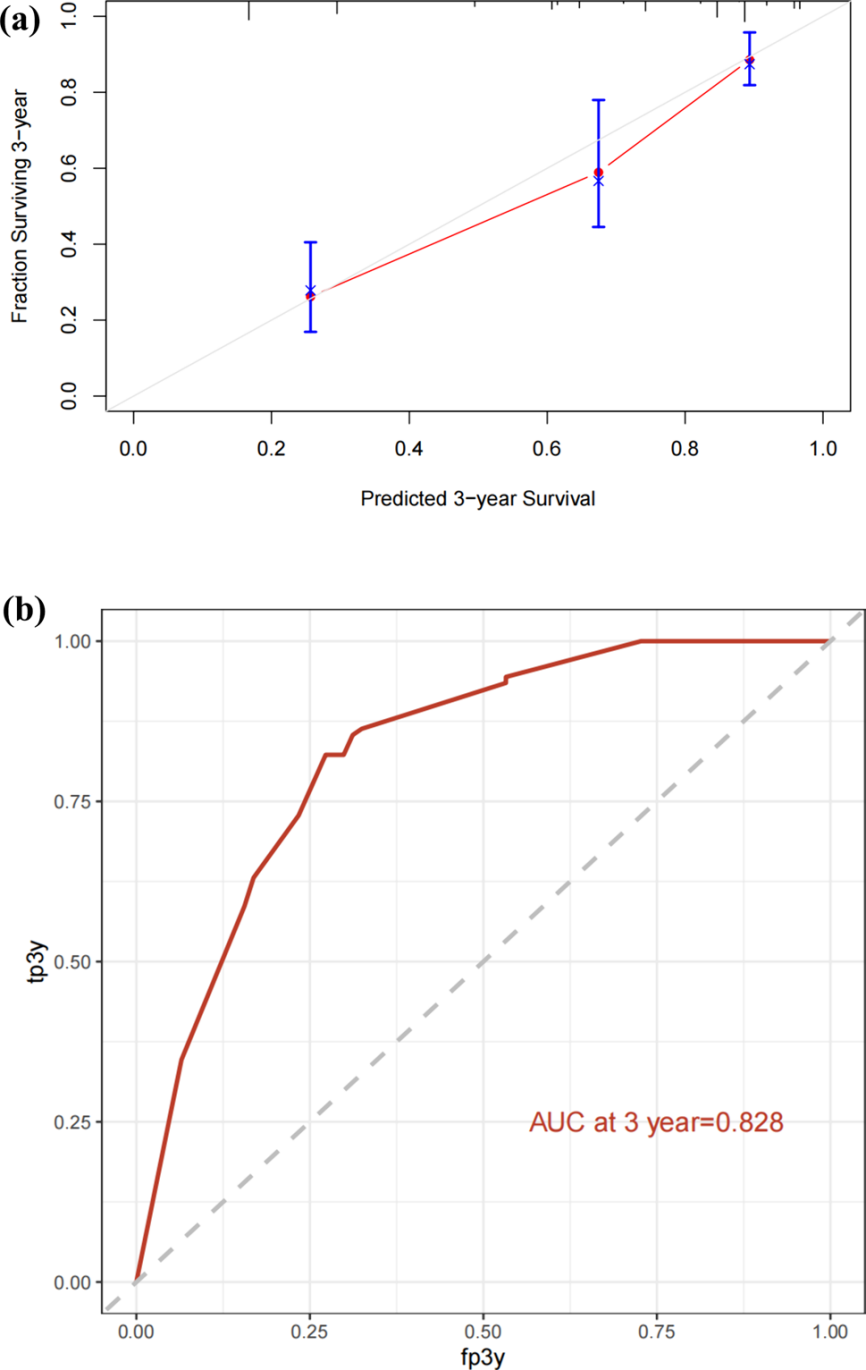
**a** Clibration curve for predicting 3-year OS of patients with advanced DLBCL in the training group. **b** The ROC curve of the nomogram model to predict the 3-year OS rate of patients with advanced DLBCL in the training group

The patients in the nomogram development cohort (*n* = 402) were divided into training cohort (*n* = 281) and validation cohort (*n* = 121) according to the ratio of 7:3. Univariate analysis was performed to identify potential prognostic factors in the training cohort: age (≥ 60 vs < 60,*p* < 0.01), BCL-2 (positive vs negative, *p* = 0.02), C-MYC (positive vs negative, *p* < 0.001), CD5 (positive vs negative, *p* = 0.003), KI-67 (high expression vs low expression, *p* < 0.001), LDH (elevated vs normal, *p* = 0.02), PLT (elevated vs normal, *p* = 0.28), FER (elevated vs normal, *p* < 0.001), β2 microglobulin (elevated vs normal, *p* < 0.001), HB (elevated vs normal, *p* = 0.04), and Alb (elevated vs normal, *p* = 0.05). In multivariate analysis, Ki-67 (*p* < 0.001), LDH (*p* = 0.05), FER (*p* < 0.001) and β2 microglobulin (*p* < 0.001) were the independent risk factors related to the prognosis of patients (Table 2). Subsequently, ki-67, LDH, FER, and β2 microglobulin were used for nomogram construction (Fig. 1). The values on the variable axis attributed to a single case were located and a vertical line was drawn upward from the variable axis to determine the total number of points assigned to the patient, enabling an estimate of the OS rate on the survival axis. Based on the constructed nomogram, the total score was used to identify three discrete risk groups according to the X-tile: low-risk, intermediate risk, and high-risk. There was no crossover in the KM curve drawn according to the risk groups scored by the nomogram. The 5-year OS rates of low-risk group, intermediate high-risk group and high-risk group were 81.6%, 44.2% and 6%, respectively. (Fig. 2). Internal validation showed good agreement between the predicted values of the nomogram and the actual 3-year OS rate in the calibration curve (Fig. 3a). In the internal validation cohort, the C-index was 0.76 and the AUC was 0.828 (Fig. 3b).

**Table 2:** Univariate and multivariate prognostic analysis of OS in patients

	Univariate		Multivariate	
	Hazard ratio (CI)	*p* value	Hazard ratio (CI)	*p* value
Sex	1.003(0.672–1.497)	0.987		
Age (≥ 60 vs < 60)	1.656(1.108–2.474)	< 0.01*	1.411(0.969–2.055)	0.072
ECOG (> 1 vs 0–1)	0.989(0.707–1.384)	0.951		
Extranodal sites (> 1 vs 0–1)	1.452(0.973–2.167)	0.067	1.030(0.709–1.497)	0.87
Bulky(positive vs negative)	1.390(0.896–2.156)	0.141		
Cell of origin (non-GCB vs GCB)	1.049(0.779–1.410)	0.75		
First-line chemotherapy regimens	1.015 (0.850–1.211)	0.87		
Assessment of efficacy	1.352(1.127–1.623)	< 0.001*	1.221(1.046–1.426)	0.01
BCL-2(positive vs negative)	1.796(1.074–3.004)	0.02*	1.118(0.757–1.846)	0.461
BCL-6(positive vs negative)	1.395(0.815–2.385)	0.22		
C-MYC(positive vs negative)	2.142(1.243–3.690)	< 0.001*	1.066(0.661–1.720)	0.793
CD10(positive vs negative)	1.253(0.807–1.944)	0.314		
CD5(positive vs negative)	1.804(1.212–2.683)	0.003*	1.112(0.763–1.623)	0.578
KI-67(> 70% vs ≤ 70%)	3.046(1.696–5.470)	< 0.001*	2.725(1.628–4.561)	< 0.001*
HBsAg status (positive vs negative)	0.877(0.497–1.546)	0.65		
LDH (abnormal vs normal)	1.589(1.064–2.372)	0.02*	1.451(0.998–2.110)	0.050*
PLT(> 100 vs ≤ 100)	0.697(0.361–1.345)	0.28		
HB(> 120 vs ≤ 120)	0.649(0.428–0.985)	0.04*	0.751(0.470–1.199)	0.23
FER(> 130 vs ≤ 130)	5.571(3.416–9.085)	< 0.001*	2.424(1.482–3.964)	< 0.001*
β2 microglobulin(> 3.05 vs ≤ 3.05)	7.097(4.350–11.580)	< 0.001*	3.301(1.987–5.482)	< 0.001*
Alb(> 40vs ≤ 40)	0.672(0.450–1.01)	0.053*	0.8668( 0.574–1.307)	0.493

*p < 0.05

### Nomogram external validation

**Fig. 4:**
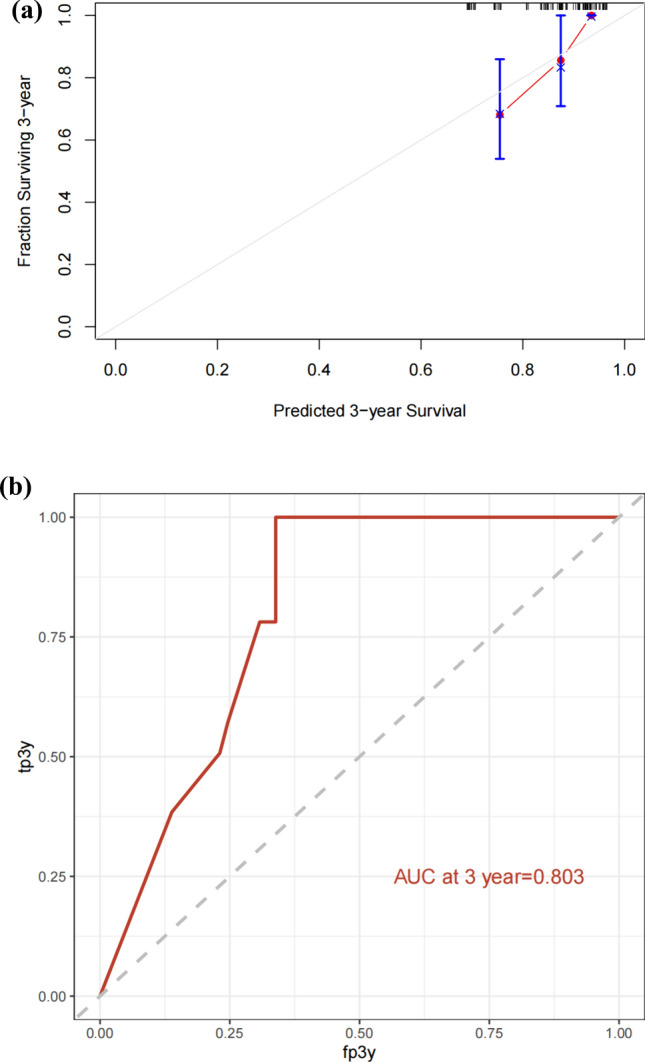
**a** Calibration curve for predicting 3-year OS of advanced DLBCL patients in validation group. **b** ROC curve of the nomogram model predicting 3-year OS of patients with advanced DLBCL in the validation group.

The nomogram was externally validated by the calibration plot in Fig. 3a and by calculating bootstrap C-index in an independent validation cohort of 121 patients. In the external validation step, the C-index of the nomogram for predicting 3-year OS was 0.74, indicating that it is a model with good discrimination. The calibration curve indicated a good nomogram (Fig. 4a), and the AUC was 0.803 (Fig. 4b).

### Comparison of OS predictive accuracy between the nomogram and current staging or prognostic scoring systems

**Fig. 5:**
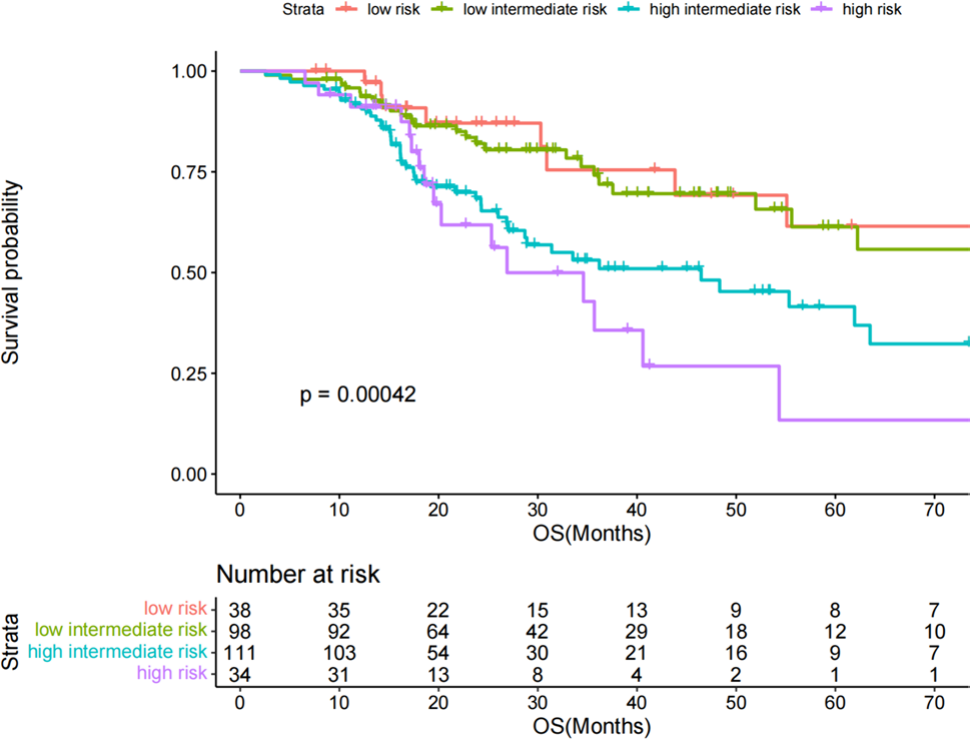
Kaplan–Meier survival curves for IPI risk groups in the training group

**Fig. 6:**
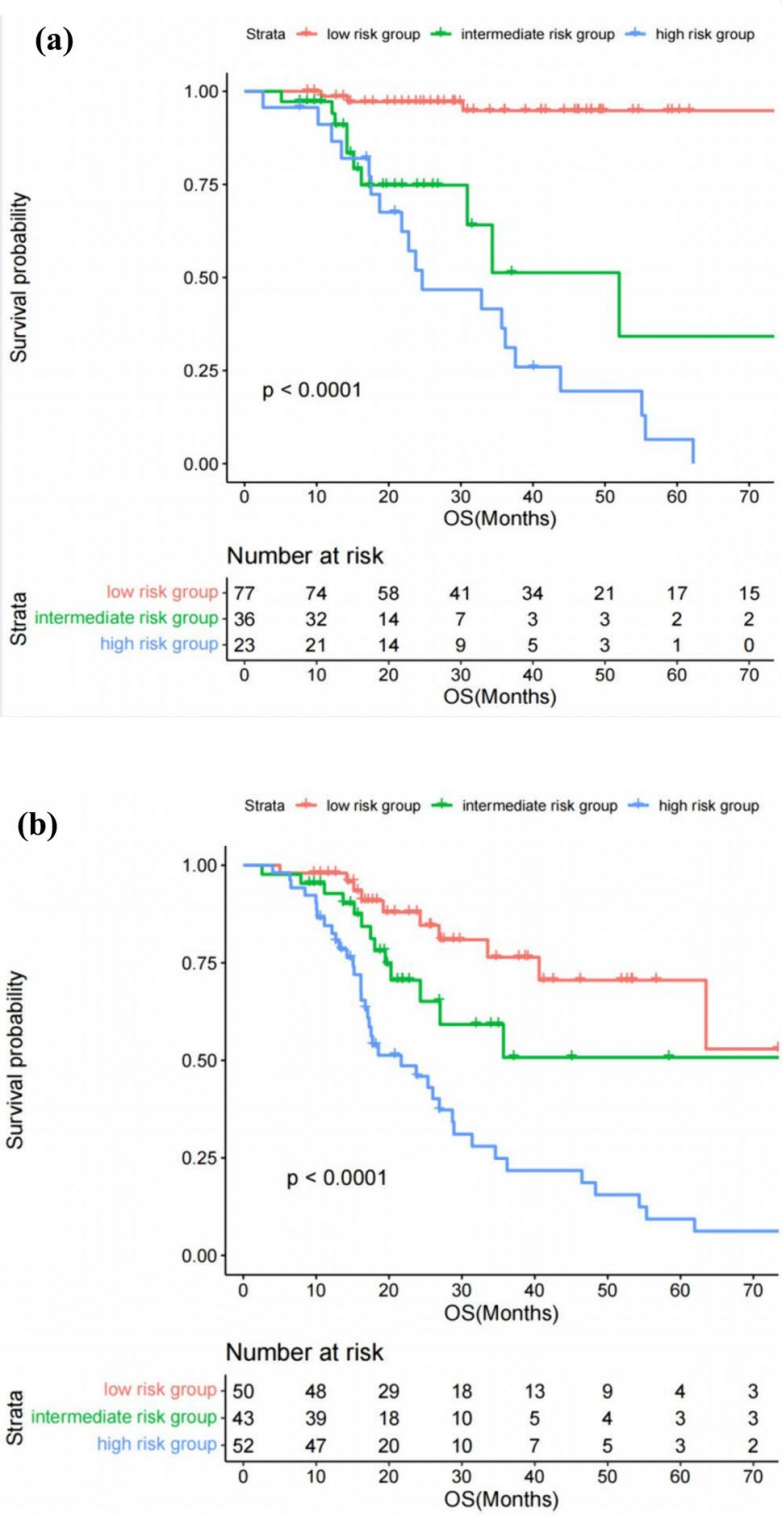
**a** The IPI low and low-intermediate risk groups in the training group were included in the Kaplan–Meier survival curve of the nomogram model. **b** The IPI high intermediate and high-risk groups in the training group were included in the Kaplan–Meier survival curve of the nomogram model

Both IPI low-risk and high-risk patients had a good prognosis stratification level (Fig. 5). For patients with stage III/IV DLBCL, the IPI score had poor stratification ability for patients with low-risk and low intermediate risk, and the 5-year survival rates were 61.5% and 55.8%, respectively. However, when the low-risk and low intermediate risk were included in our nomogram, the nomogram showed better stratification ability, and the 5-year survival rates were 93%, 34% and 6%, respectively (Fig. 6a). Similarly, for patients with stage III/IV DLBCL, the IPI score had poor stratification ability for high-intermediate and high-risk groups, with 5-year survival rates of 36.9% and 13.4%, respectively. However, when high-intermediate and high-risk groups were included in our nomogram, the nomogram showed better stratification ability. The 5-year survival rates of low, intermediate and high-risk patients were 52.9%, 30% and 6% (Fig. 6b), respectively. The C-index of the nomogram in the training cohort (0.72) was higher than that of the IPI (0.70).

## Discuss

DLBCL is the most common type of non-Hodgkin lymphoma, with strong heterogeneity and poor prognosis [23]. About 60–70% of DLBCL patients are at stage III/IV at the time of initial diagnosis. Since the 1990s, IPI has become a standard tool for risk stratification and guiding therapy [24]. In the IPI score, stage is an important prognostic factor, and the higher the stage, the worse the prognosis. However, for advanced DLBCL patients with poor prognosis, IPI does not seem to show good prognostic stratification ability. At present, there is no prognostic tool for advanced-stage DLBCL. Therefore, the aim of this study is to develop a good prognostic model for advanced-stage DLBCL patients to accurately guide treatment.

The nomogram was designed to estimate the probability of 1, 3, and 5-year OS based on multivariate Cox proportional hazards model. The final nomogram model consisted of four variables from routine clinical practice: Ki-67, LDH, FER and β2 microglobulin. These four factors have been confirmed to have an impact on the prognosis of DLBCL patients in other studies. For Ki-67, it has been shown that high Ki67 index is an important adverse prognostic factor in DLBCL patients after the introduction of rituximab [25, 26]. For LDH, the standard prognostic score IPI has included LDH in the prognosis stratification of DLBCL patients, and high LDH indicates poor prognosis [18, 27].For β2 microglobulin, some studies have shown that β2 microglobulin can identify subsets with poor prognosis in intermediate-risk patients with DLBCL [28] and may improve NCCN-IPI score [29]. For ferritin, some studies have shown that the prognostic model incorporating ferritin has better prognostic stratification ability than IPI for DLBCL [30]. The DLBCL patients were scored according to the nomogram, and the risk group was divided according to the total score. According to the prognosis survival curve, the model had a good predictive function.

In IPI score, stage was an important prognostic factor, and stage III/IV accounted for 1 point [31]. The efficacy of IPI score in the stratification of prognosis will be affected. Our study shows the same conclusion. The total score of the nomogram was divided into three risk groups by X-tile software. There was no crossover in the KM curve drawn according to the risk groups scored by the nomogram, suggesting that the nomogram model has good prognostic discrimination ability in patients with advanced DLBCL. However, the prognosis stratification level of patients with low-risk and low-intermediate, high-intermediate and high-risk according to IPI score was poor, but the low-risk and low-intermediate, high-intermediate and high-risk groups were included in the nomogram for prognosis stratification, and the nomogram showed good prognosis stratification level. Therefore, our nomogram showed better prognostic stratification ability than IPI score for patients with advanced DLBCL.

This study still has several limitations. First, there is no uniform standard treatment for DLBCL patients in this study, which has R-CHOP, R-DA-EPOCH, or R-CHOP + X, which may cause some impact on the study. Second, this study was retrospective, which may have caused potential selection bias. Third, because there were some missing values of some indicators in this database, these indicators were not included in the construction of the model.

In conclusion, we have developed and externally validated a nomogram that can predict 5-year OS in advanced DLBCL in a highly accurate manner based on a large cohort of affected patients from endemic areas. The proposed nomogram showed a better discrimination level than IPI in the training set and provided individual risk assessment for DLBCL patients.

## Data Availability

The data set used and/or analyzed during the current study is available from the corresponding author on reasonable request.
